# Bone sialoprotein does not interact with pro-gelatinase A (MMP-2) or mediate MMP-2 activation

**DOI:** 10.1186/1471-2407-9-121

**Published:** 2009-04-22

**Authors:** Queena Hwang, Sela Cheifetz, Christopher M Overall, Christopher A McCulloch, Jaro Sodek

**Affiliations:** 1 CIHR Group in Matrix Dynamics, University of Toronto, Toronto, Canada; 2 CIHR Group in Matrix Dynamics, University of British Columbia, Vancouver, Canada

## Abstract

**Background:**

A recent model for activation of the zymogen form of matrix metalloproteinase 2 (MMP-2, also known as gelatinase A) has suggested that interactions between the SIBLING protein bone sialoprotein (BSP) and MMP-2 leads to conformational change in MMP-2 that initiates the conversion of the pro-enzyme into a catalytically active form. This model is particularly relevant to cancer cell metastasis to bone since BSP, bound to the αvβ3 integrin through its arginine-glycine-aspartic acid motif, could recruit MMP-2 to the cell surface.

**Methods:**

We critically assessed the relationship between BSP and proMMP-2 and its activation using various forms of recombinant and purified BSP and MMP-2. Gelatinase and collagenase assays, fluorescence binding assays, real-time PCR, cell culture and pull-down assays were employed to test the model.

**Results:**

Studies with a fluorogenic substrate for MMP-2 showed no activation of proMMP-2 by BSP. Binding and pull-down assays demonstrated no interaction between MMP-2 and BSP. While BSP-mediated invasiveness has been shown to depend on its integrin-binding RGD sequence, analysis of proMMP-2 activation and the level of membrane type 1 (MT1)-MMP in cells grown on a BSP substratum showed that the BSP-α_v_β_3 _integrin interaction does not induce the expression of MT1-MMP.

**Conclusion:**

These studies do not support a role for BSP in promoting metastasis through interactions with pro-MMP-2.

## Background

Bone sialoprotein (BSP) is a highly glycosylated and sulfated phosphoprotein that is expressed largely in mineralizing tissues [1] but is also associated with cancer metastasis. Elevated levels of BSP have been reported in tumors and serum from patients with breast, lung, prostate, or thyroid cancer [2]. Expression of BSP in cancer has been associated with metastasis of tumor cells to bone [3] as well as hydroxyapatite crystal formation in tumor tissues and breast cancer cell lines [4].

Matrix metalloproteinases (MMPs) are a family of zinc-dependent endopeptidases that cooperate to modulate homeostasis of the extracellular environment by regulating oncogenic signaling networks and degrading extracellular matrix components, thereby contributing to tumor cell progression [5-7]. MMP-2 (also known as gelatinase A) is made up of five structural domains including an inhibitory pro-domain [8-10]. Functional activity is regulated by enzymatic removal of the inhibitory pro-domain. A primary mechanism of proMMP-2 activation involves formation of a tri-molecular complex on cell surfaces involving tissue inhibitor of metalloproteinase-2 and membrane type 1-MMP (MT1-MMP) [11,12]. An alternative mechanism for controlling MMP-2 activity has invoked apparent structural changes that arise from specific interactions between BSP and proMMP-2 [13,14]. It was reported that upon binding to BSP the proteolytic activity of proMMP-2 increased significantly, but paradoxically without removal of the inhibitory pro-peptide [13]. It was suggested that BSP-mediated conformational changes upon partnering with proMMP-2 may facilitate removal of the inhibitory pro-peptide by another protease, which is similar to the binding and activation of proMMP-2 by MT1-MMP. A 26 amino acid domain of BSP appears to be involved in the displacement of MMP-2's propeptide from the active site of MMP2, thereby enhancing protease activity [14].

Since BSP and MMP-2 are associated with tumor progression [2,15-17,7], the potential modulation of proMMP-2 activity by BSP is particularly relevant to tumor metastasis. We critically assessed potential interactions between BSP and proMMP-2 that mediate proMMP-2 activation.

## Methods

### Reagents

Recombinant proMMP-2 was produced as described [18]. Bacterial recombinant rat BSP, rat native BSP, and BSP fragments were produced by Harvey Goldberg (University of Western Ontario). The BSP fragments contained amino acids 1–100, 99–200, 200–301, 51–150, and 99–250) of BSP. Recombinant human BSP expressed in human bone marrow stromal cells was from N.S. Fedarko (Johns Hopkins). Porcine BSP, G2 BSP, human BSP, and pig OPN were purified from 0.5 M EDTA and 4 M guanidine-HCL (G2) extracts of bone tissues. OPN was purified from bovine milk.

### Cell Culture

Human breast cancer cell lines MDA-MB231, MCF7, T47D, and fibrosarcoma HT1080 cells were obtained from ATCC. Rat bone marrow stromal cells were from S. Pitaru (Tel Aviv, Israel). Human gingival fibroblasts were grown in primary culture and for production of activated MMP2, cells were treated with concanavalin A. Cells were maintained in α-minimum essential medium (MEM) containing 10% fetal bovine serum. T47D cells were maintained in a monolayer culture in RPM1 containing 1% Glutamax 1 and10% FBS.

### Cell Culture on BSP substratum

Cells were seeded on to 24-well plates coated with 30 nM rat recombinant or rat native BSP. For analysis of proMMP-2 activation, conditioned medium was collected after 24 hours in serum-free medium, concentrated, and analyzed for gelatinase activity by zymography. To analyze MT1-MMP mRNA, cells were seeded at 1.0 × 10^6 ^cells/mL on a non-tissue culture 96-well ELISA plates coated with rat native BSP (0.15 μM) or poly-L-Lysine (0.1%).

### Tryptophan fluorescence binding assay

Since BSP contains no tryptophans, the binding of BSP to proMMP-2 was measured from the shift in tryptophan fluorescence of proMMP-2 (15 tryptophan residues) (excitation = 295 nm; emission = 300–400 nm) after addition of BSP, BSP peptides, or control proteins (from 17 nM-1165 nM) to proMMP-2 (333 nM). All spectra were corrected for buffer and dilution effects. Under these conditions, fluorescence observed was attributed exclusively to tryptophans from proMMP-2 as described previously [13]. To estimate dissociation constants (K_d_), a saturation curve for BSP-proMMP-2 complex formation was obtained. *K*_*d *_values were calculated using the Scatchard equation *r/[free BSP] *= *n/K*_*d *_- *r/K*_*d *_where *n *represents the number of binding sites and *r *= *[bound BSP]/[total proMMP-2]*. Each experiment was carried out in triplicate.

### Analysis of proMMP-2 auto-activation

Gelatinase activities were determined by gelatin zymography [19]. BSP-mediated proMMP-2 activation was monitored by incubating 0.2 ng/2 μL of BSP with proMMP-2 (0.05, 0.2, 0.5, or 2 ng) and adding collagenase assay buffer[20] (total volume of 8 μL). For positive controls, activated MMP-2 was obtained from concanavalin A-treated fibroblast-conditioned medium. After 4 hours of incubation at 21°C, samples extracted in SDS-PAGE sample buffer (without DTT) were analyzed by gelatin zymography.

### Gelatinase substrate assay

ProMMP-2 activity was measured with a highly quenched, fluorescein-labeled (DQ) gelatin substrate at 21°C. Upon proteolytic digestion, its fluorescence is revealaed and can be used to measure enzymatic activity. Each assay was conducted at 21°C in collagenase assay buffer. ProMMP-2 (1.4 nM or 2.8 nM) was added to 12.5 μg/mL substrate in the presence or absence of BSP (4.9 nM or 9.8 nM). Cleavage of the substrate was monitored using a micro-plate based multi-detection reader (485 nm excitation, 520 nm emission filters; FLUOstar OPTIMA, BMG Labtech, Offenburg, Germany). Changes in fluorescence intensity were monitored in relation to controls: substrate + BSP, substrate or proMMP-2 as negative controls, and substrate + proMMP-2 activated with aminophenylmercuric acetate (APMA) as a positive control.

### Binding assays

For analysis of bound and unbound proMMP-2, 25 ng/50 μL of the pro-enzyme was added to ELISA plates coated with various concentrations of pBSPE (porcine BSP-extract), pBSPG2 (G2-extract), human bone proteins (hBP), pig bone OPN (OPN), and incubated for 1 hour at 21°C. Supernatants and bound proteins were analyzed by gelatin zymography. For analysis of potential adaptor molecules, conditioned medium from cells was added to ELISA plates coated with 40 nM rat recombinant BSP, BSA, or gelatin and incubated for 1 hour at 21°C. Supernatant and bound proteins extracted with sample buffer were analyzed for gelatinase activity by zymography. For analysis of potential adaptor molecules, 200 μL of conditioned medium collected from MDA-MB231, rat bone marrow stromal cells, HT1080 cells, and human gingival fibroblasts at 60 hours after seeding was added to a 96-well ELISA plated coated with 40 nM rat recombinant BSP, BSA or gelatin. Each mixture was incubated for 1 hour at 21°C. Supernatant and bound proteins extracted with sample buffer were analyzed for gelatinase activity using zymography.

### Biotinylation of bone proteins

For biotinylation, 22 moles of biotin were used per mole of bone proteins. Correspondingly, appropriate amount s of biotin (1 mg of biotin dissolved in mL DMSO) were added to each protein preparation. The mixtures were stirred for 2 hr at 4°C. To remove free biotin, the mixtures were desalted on a 10 mL desalting column equilibrated in 50 mM ammonium bicarbonate buffer, pH 8.5. Biotinylation of the eluate fractions was assessed using dot blot analysis, where 2 μL of each fraction was taken and probed with streptavidin horseradish peroxidase. Finally, the highly biotinylated fractions were pooled, speed vacuumed and reconstituted in water.

### Solution phase binding assay

Biotinylated BSP was utilized to examine the potential interaction between BSP and proMMP-2 in solution and in these experiments 25 ng proMMP-2 was incubated with 5 μg biotinylated protein in 50 μL Tris-Tween (0.05%; pH ~7.6) for 1 hour at 21°C. To isolate BSP along with bound proteins, streptavidin beads were added, incubated for 30 minute at 21°C, centrifuged, and supernatants were collected. Beads were rinsed and supernatants and bead eluates were analyzed by gelatin zymography. Controls included no MMP-2 and no BSP.

### Real-time PCR

RNA was extracted from cells using a Stratagene RNA miniprep kit. Total RNA (1 μg) was reverse transcribed and real-time PCR for MT1 was performed using the TaqMan^® ^Gene Expression Assay system using validated probes human MT1-MMP (no. 4331182) and eukaryotic 18S endogenous control (no. 4319413E).

### Statistical analysis

All assays were repeated at least 3 times in 3 separate experiments. For data involving continuous variable, the means and standard errors of the mean were calculated and where appropriate, analysis of variance was used to examine differences between multiple groups.

## Results

### BSP induces non-specific quenching of proMMP-2

**Figure 1:**
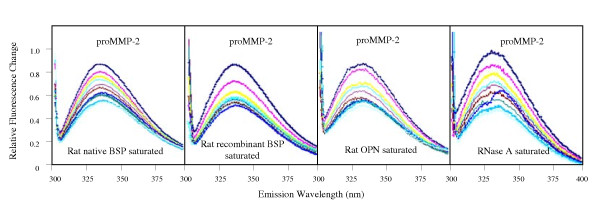
**Fluorescence emission spectrum of BSP-treated proMMP-2**. proMMP-2 (333 nM) was incubated with increasing concentrations of native or recombinant BSP, OPN or RNase A (negative controls). Emission scans were obtained after each addition of BSP (excitation wavelength of 295 nm). In all cases, titrations of proMMP-2 yielded proportional quenching of the proMMP-2 tryptophan emission spectra.

Due to variations of BSP phosphorylation of serines and O- and N-linked glycosylation, recombinant or native rat BSP (purified from long bones of adult rats) were used in binding studies to assess binding between proMMP-2 and BSP. Intrinsic tryptophan fluorescence measurements demonstrated that titration of proMMP-2 with BSP resulted in a proportional quenching of MMP tryptophan emission spectra (Fig. 1), suggestive of direct protein-protein interactions. Since proMMP-2 contains 15 tryptophan residues, whereas BSP contains none, the quenching of the tryptophan fluorescence signal suggests that proMMP-2 undergoes significant conformational changes, exposing internal tryptophan residues to a more polar environment in the presence of BSP with an apparent K_d _of 0.27 ± 0.11 μM. However, control studies using osteopontin and RNase A in the same system also yielded a similar quenching of the proMMP-2 tryptophan emission spectra as well as the derivation of similar K_d _values.

**Figure 2:**
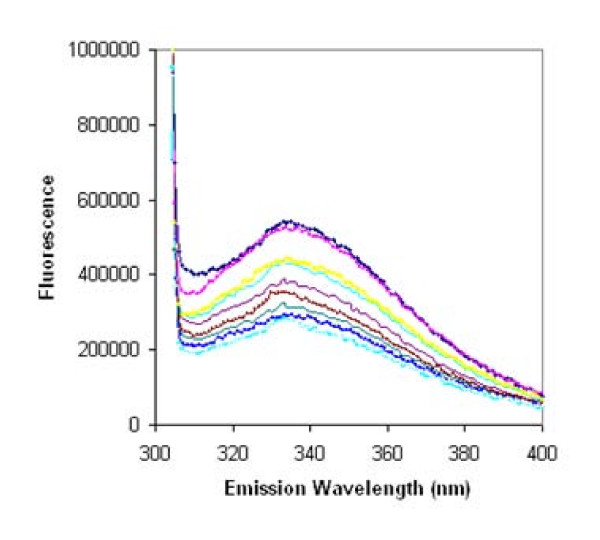
**Tryptophan fluorescence profile**. proMMP-2 (333 nM) was incubated with nM amounts of native BSP. Emission scans were obtained after each addition of BSP (excitation wavelength = 295 nm). Emission peak was at 335 nm.

**Figure 3:**
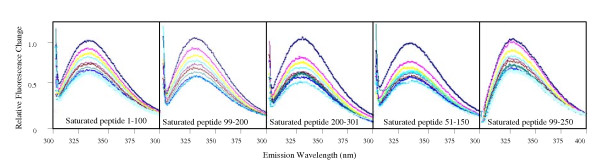
**Interactions between BSP peptides and proMMP-2**. proMMP-2 (333 nM) was incubated with nM amounts of BSP peptides. Emission scans were obtained after each addition of BSP (excitation wavelength = 295 nm). Emission spectra show that titration of proMMP-2 with each BSP peptide yielded proportional quenching of the proMMP-2 emission spectra.

The human recombinant BSP that was used previously to detect binding between BSP and proMMP-2 [13] may have included modifications necessary for measurement of potential interactions. Accordingly, the effect of post-translational modifications on the proposed interaction between BSP and MMP-2 was investigated using human recombinant BSP obtained from N. Fedarko (Fig. 2). The emission peak in intrinsic fluorescence was observed at ~335 nm, which is in contrast to the previous study [13] that reported an emission peak at 360 nm and an interaction between proMMP-2 and BSP with a k_d _in the nanomolar range. When the MMP-binding site within BSP was studied by intrinsic fluorescence using BSP peptides (Fig. 3), each BSP fragment showed quenching of the MMP-2 tryptophan fluorescence signal, similar to the emission spectra obtained using the full-length BSP molecule and the control proteins suggesting non-specific interactions.

### BSP does not modify proMMP-2 activity

**Figure 4:**
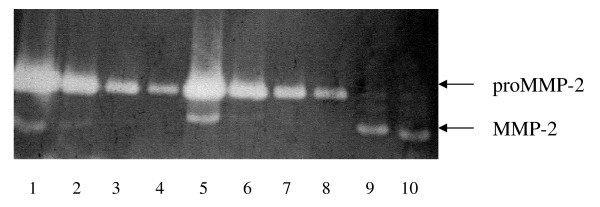
**Zymography analysis of BSP-treated proMMP-2**. ProMMP-2 was incubated with (lanes 5–8) or without (lanes 1–4) increasing amounts of BSP for 4 hours at 21°C, and resolved by zymography. ConA cell-activated MMP-2 were used as standards (lanes 9–10). Lane 1, 2 ng proMMP-2; lane 2, 0.5 ng proMMP-2; lane 3, 0.2 ng proMMP-2; lane 4, 0.05 ng proMMP-2; lane 5, 2 ng proMMP-2 + 2 ng BSP; lane 6, 0.5 ng proMMP-2 + 2 ng BSP; lane 7, 0.2 ng proMMP-2 + 2 ng BSP; lane 8, 0.05 ng proMMP-2 + 2 ng BSP; lanes 9 and 10, 0.05 ng conA activated MMP-2.

To examine potential activation induced by the addition of BSP to proMMP-2, zymography was employed to estimate the amount of mature enzyme of smaller molecular weight (59 or 62 kDa). In concentrations where BSP is in excess of proMMP-2, there was no evidence for significant removal of the pro-domain (Fig. 4) although in positive controls, proMMP-2 that had been activated in concanavalin A-treated cells showed lower molecular mass MMP-2 (Fig. 4, lanes 9, 10), consistent with cleavage of the pro-peptide and enzyme activation. When zymography bands were further assessed, each BSP-treated proMMP-2 sample resulted in an identical migration pattern as that of untreated enzyme. This is consistent with previous findings indicating that BSP binding does not induce significant cleavage of the pro-peptide [13]. Therefore, BSP-treated proMMP-2 migrates as an intact molecule (*Mr *of ~66 kDa) on zymograms since the pro-peptide remains attached.

**Figure 5:**
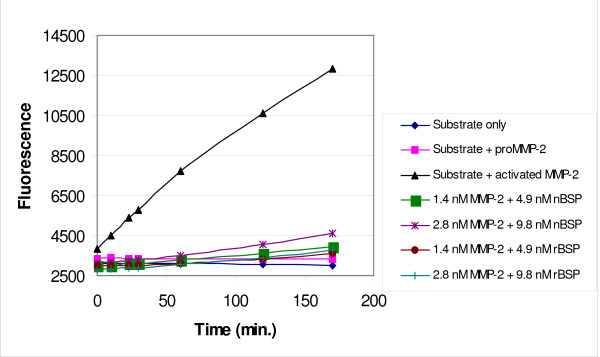
**ProMMP-2 activity after incubation with BSP**. ProMMP-2 (1.4 or 2.8 nM) was incubated with recombinant BSP (rBSP) or native BSP (nBSP) (4.9 or 9.8 nM) and 12.5 μg/mL fluorescent substrate. Results are values calibrated with fluorescence from substrate + BSP controls. Fluorescence levels of other controls, including substrate only, substrate + proMMP-2 and substrate + APMA-activated enzyme, are also shown.

The effect of BSP on proMMP-2 activity was examined using fluorescent labeled gelatin substrate. Treatment of proMMP-2 with increasing concentrations of recombinant BSP or fetal porcine BSP did not alter enzymatic activity compared to latent enzyme alone (Fig. 5). Using the same substrate, the ability of OPN to activate proMMP-3 was assayed, but activity above control values was also not observed. Since BSP may interact with proMMP-2 so that the inhibitory pro-peptide is removed from the active site [13], hence exposing the active site, we considered that the presence of BSP would lead to significant cleavage (auto-activation) to the lower molecular weight, active MMP-2. However, we found no increase in the amount of pro-peptide-free MMP-2 by zymography confirming the fluorescent gelatin cleavage assays. Further, BSP did not mediate proMMP-2 catalytic activity as shown with the fluorescent substrates.

### Analysis of bound and unbound MMP-2

**Figure 6:**
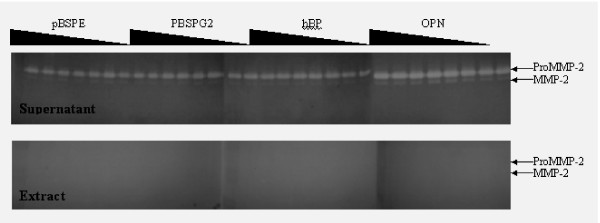
**ProMMP-2 recovery in BSP-unbound sample**. ProMMP-2 (0.5 mM) was added to decreasing concentrations of indicated SIBLING proteins (40 mM, 20 mM, 10 mM, 5 mM, 2.5 mM, 1.2 mM, 0.6 mM, and 0 mM) coated on an ELISA plate, and incubated at 21°C for 4 hours. Samples of the unbound (Supernatant) and bound (Extract) proteins were extracted in SDS sample buffer, and analyzed by zymography. proMMP-2 was recovered completely in the latent form in the unbound (Supernatant) fractions.

Despite the lack of significant pro-peptide cleavage when proMMP-2 dose response curves to BSP were examined, we hypothesized that BSP-induced activation might involve only a fraction of the total amount of enzyme. We used ELISA plates to resolve BSP-bound and unbound fractions, which allows for higher resolution examinations of the BSP-proMMP-2 interactions. Previous findings have suggested a 1:1 stoichiometry of binding between BSP and proMMP-2 and a K_d _value of 2.9 ± 0.9 nM [13]. Such a strong affinity should allow for detection of the interaction. However, our data showed no binding between the proMMP-2 and BSP as detected when the BSP-bound (extract) and unbound (supernatant) fractions were analyzed by zymography (Fig. 6).

We considered that the lack of association between proMMP-2 and BSP could be a consequence of disruption of a binding motif from fixing BSP to a hydrophobic surface. Accordingly, we assessed the ability of BSP to associate with proMMP-2 in solution. Bone proteins were biotinylated, incubated with proMMP-2 and isolated using streptavidin beads. When bead-bound entities were assessed by zymography, each bead-purified bone protein showed no evidence of MMP-2 binding (data not shown). Further, MMP-2 was recovered entirely in the latent form (*M*_*r *_of 66 kDa) in the supernatant. Alternatively, when biotinylated bone proteins were pre-bound to streptavidin beads, followed by the addition of proMMP-2, similar results were observed.

### Analysis of potential adaptor molecules

**Figure 7:**
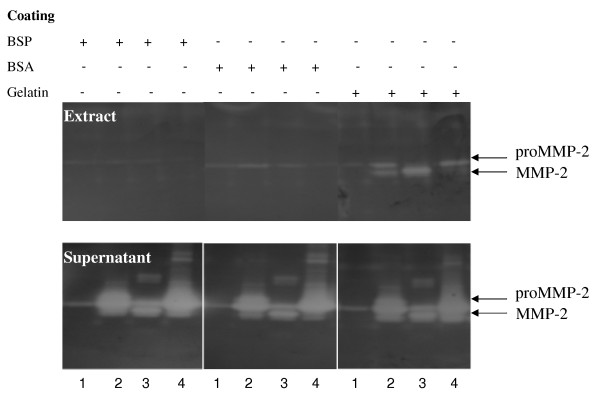
**MMP-2 from conditioned medium recovery in BSP-unbound fraction**. Serum-free conditioned media collected from: 1) MDA-MB231, 2) rat bone marrow cells, 3) HT1080, and 4) human gingival fibroblasts were added to ELISA plates coated with indicated proteins (35 μM) and incubated at RT for one hour. Samples of bound (extract) and unbound (supernatant) proteins were extracted in SDS sample buffer and analyzed by zymography. Zymography shows that when MMP-2 is added to BSP-coated plates, both latent and active enzymes are recovered completely in supernatants. BSA and gelatin were used as negative and positive MMP-2-binding controls respectively.

Because of the lack of any evidence of specific binding of proMMP-2 to BSP, the need for potential adaptor molecules in this interaction was examined using solid phase binding assay on ELISA plates followed by zymography. Serum-free conditioned medium collected from MDA-MB231, rat bone marrow cells, HT1080 or human gingival fibroblasts were used as a source of MMP-2 and added to BSP that was conjugated to an ELISA plate. Since the reported binding between BSP and proMMP-2 was initially identified by a co-purification of proMMP-2 and recombinant BSP expressed in bone marrow cells [13], we hypothesized that given the absence of a direct interaction then complexes with other proteins might be required, similar to the TIMP-2 bridge between the physiological activator MT1-MMP and MMP-2 [18]. Nonetheless, zymography analysis of BSP-bound (extract) and unbound (supernatant) fractions revealed that latent and active MMP-2 secreted by bone marrow cells (Fig. 7), as well as the other cell lines, were recovered entirely in the supernatant, unbound fraction as observed for recombinant proMMP-2.

### ProMMP-2 activation is unaffected by cellular adhesion to BSP

**Figure 8:**
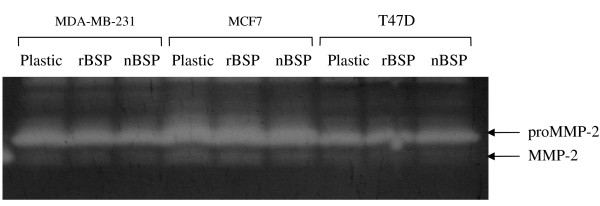
**Effect of cell attachment to BSP on MT1-MMP-mediated activation of proMMP-2**. Serum-free conditioned medium was collected from the indicated breast cancer cell lines seeded on BSP (30 μM) coated on ELISA plates, concentrated, and analyzed on zymograms. There were no significant differences in the level of proMMP-2 activation between cells grown on recombinant (r)BSP or native (n)BSP compared to cells grown on plastic.

Despite the lack of direct or indirect interaction observed between BSP and proMMP-2, clustering of the α_2_β_1 _integrins in cancer cells stimulated by fibrillar collagen has been shown to promote tyrosine kinase-mediated events that result in expression of MT1-MMP and proMMP-2 activation [21]. To investigate the consequences of integrin α_v_β_3 _clustering by BSP, the levels of proMMP-2 activation in MDA-MB231, MCF7, and T47D cells grown on BSP substrata were compared to that of cells grown on plastic. There was a similar level of proMMP-2 activation in cells after attachment to BSP in comparison to cells grown on plastic (Fig. 8). Since proMMP-2 activation is directly associated with the level of MT1-MMP activity, these results indicated that cellular binding to BSP via integrin α_v_β_3 _does not modify MT1-MMP activity on the cell surface. Previously we have shown that proMMP-2 does not directly bind α_v_β_3_[22].

### Cellular adhesion to BSP does not alter MT1-MMP transcript level

**Figure 9:**
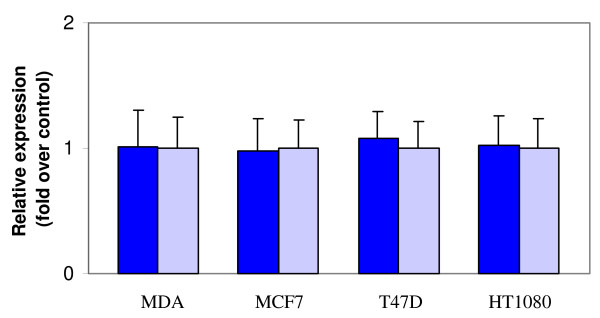
**MT1-MMP transcript levels after BSP stimulation**. MDA, MCF7, T47D, or HT1080 cells were seeded on native BSP (blue bars) or poly-L-Lysine (grey bars) coated on an ELISA plate. Total RNA was reverse transcribed and subjected to qPCR analysis using specific primers for MT1-MMP. Results were normalized as fold increase over cells seeded on poly-L-Lysine and expressed as mean ± SEM (n = 3). From the comparison no significant differences (p > 0.2) in the MT1-MMP transcript level were observed between cells grown on BSP and cells grown on poly-L-Lysine.

Since the activity of MT1-MMP is regulated at multiple steps, differences in MT1-MMP expression may not be detected by analysis of proMMP-2 activation. Accordingly, MT1-MMP mRNA levels were analyzed by real time RT-PCR to investigate quantitatively whether MT1-MMP mRNA levels are different between cancer cells grown on a BSP substratum and on poly-L-Lysine. Real-time PCR results (Fig. 9) did not detect any significant changes in the MT1-MMP transcript level by stimulation with BSP (p > 0.2), which was consistent with an unaltered level of MT1-MMP activity as observed by zymography.

## Discussion

Cellular invasion in metastasis is a coordinated event that involves multiple metabolic processes and cellular components, including deployment and activation of cell adhesion molecules and proteolytic enzymes. Frequently, multimers of proteases show increased catalytic efficiency and in the plasma membrane, enabling focal proteolysis under cellular control. MMPs have traditionally been associated with tumor cell invasion and metastasis, in particular MMP-2 and its activator MT1-MMP. MMP-2 is uniquely activated on the cell surface by MT-MMPs in a highly regulated process after complex formation of pro- and active MMP-2 with MT1-MMP and TIMP-2 [23,12]. Extracellularly, clustering by heparin or ConA [24] and claudin [25], increases MMP-2 activation. Recently, specific interactions between BSP and latent forms of MMP-2 have been reported that resulted in activation of proMMP-2 [13]. We assessed here the ability of various forms of BSP to bind and activate proMMP-2. We investigated the possibility that BSP activated proMMP-2 by analysis of gelatinase activity using a fluorescent substrate, but the analysis showed no activation of proMMP-2. Further, when OPN, another SIBLING protein, was assessed in proMMP-3 activation using the same substrate, no activation could be detected. Therefore, BSP does not appear to be involved in the activation of proMMP-2.

After careful examination of the conditions used for activation in the previous study [13], we noticed that despite a reported K_d _value of 2.9 ± 0.9 nM, a 500-fold molar excess of BSP was necessary to demonstrate proMMP activation. We repeated these experiments using the human BSP at this same ratio but again found no activation. Given the potential ability of BSP to promote displacement of pro-peptides from active sites of proMMP-2 [13], we considered that there may be auto-activation of the latent enzyme in the presence of BSP. However, when proMMP-2 was treated with BSP, the proMMP-2 migrated as an intact molecule on zymograms, indicating that BSP does not activate proMMP-2.

Activation of MMP-2 requires unidentified protein-protein interactions, one of which might involve BSP. Extracellularly, one of the known interactors is native type I collagen, which results in the lateral association of MT1-MMP to accelerate activation of progelatinase A [26,27]. As a result of our inability to detect BSP-induced activation of proMMP-2, we examined the interaction of these two proteins using binding assays. Since previous findings [13] have suggested a 1:1 stoichiometric binding between BSP and proMMP-2 with a K_d _value in the low nanomolar range, such an affinity presumably allows detection of the interaction using less sensitive assays such as affinity adsorption. However, we found no evidence of interaction between BSP and proMMP-2 using these assays.

To address the possibility of cell-derived adaptor molecules required for the BSP-proMMP-2 interaction, BSP was incubated with conditioned medium collected from breast cancer cells, bone marrow cells or human gingival fibroblasts. As observed for recombinant proMMP-2, latent and active MMP-2 secreted by cancer cells also did not bind to BSP. Notably, BSP is highly heterogeneous as a result of variations in the phosphorylation of serines and O- and N-linked glycosylation [28]. Presumably, BSP expressed by diverse cells types is modified differently, and variations in post-translational modifications may determine the activity of these proteins and the binding and activation of proMMP-2. Accordingly we employed recombinant BSP, BSP purified from bone, or recombinant human BSP to assess binding to proMMP-2. As we were unable to detect binding of any of the BSPs to proMMP-2, there is evidently a need to re-assess the potential ability of BSP to bind to and activate proMMP-2 in the context of cancer cell metastasis although we cannot rule out the possibility that much more highly glycosylated BSP than the bovine BSP we used here could conceivably mediate an interaction with proMMP2.

MMP-2 binds to the surface of cancer cells via the fibronectin type II module repeats of the enzyme [29,30]. Despite the lack of an interaction between BSP and proMMP-2, it is possible that an interaction between BSP and the α_v_β_3 _integrin itself may trigger downstream signaling events that affect the expression, processing, and activity of MMP-2. Thus, the requirement of an active RGD sequence in BSP-mediated cancer cell invasion suggests that BSP binding to the α_v_β_3 _integrin may promote clustering of integrin molecules, which could activate downstream signaling events. Notably, ECM proteins can promote raft formation and type I collagen activates MMP-2 through β_1_-integrins, which increases MT1-MMP levels [21], and by direct binding of pericellular native type I collagen with the MT1-MMP hemopexin domain [26]. MT1-MMP enhances focal proteolysis [31] and experimental metastasis [32], is associated with MMP-2 activation in lung carcinoma [33] and invasive human breast cancer cell lines [34,35], and is over-expressed in high-grade gliomas, fibrosarcomas [36] and in carcinomas of the lung, stomach, head and neck [37]. However, in our studies there was no evidence of integrin-mediated enhancement in the level of MT1-MMP transcript level, nor in MT1-MMP activity. Evidently, a more complete understanding of integrin-mediated signaling events will be important for defining the significance of BSP binding to the α_v_β_3 _integrin *in vivo*.

## Conclusion

Collectively, using the methods reported here, our studies do not support a role for BSP in promoting pro-MMP-2 activation.

## Competing interests

The authors declare that they have no competing interests.

## Authors' contributions

QYJH conducted the experiments and the analyses and wrote the first drafts of the manuscript. SC designed the RT-PCR experiments and probes. CMO designed the proMMP2 activation experiments and contributed to the penultimate draft manuscript. CAM drafted the manuscript and wrote the final draft. JS designed the experiments and helped to write the initial drafts.

## Pre-publication history

The pre-publication history for this paper can be accessed here:

http://www.biomedcentral.com/1471-2407/9/121/prepub
